# Lack of evidence for a consistent differential impact of tail and tunnel handling on markers of welfare in laboratory mice

**DOI:** 10.1038/s41598-025-07384-w

**Published:** 2025-07-01

**Authors:** Neele Meyer, Rebekka Gottschalk, Lena Jakobi, Anna-Maria Schönhoff, Chadi Touma

**Affiliations:** Department of Behavioural Biology, Osnabrück University, Osnabrück, Germany

## Abstract

Different handling methods for laboratory mice have been intensely debated in light of refining animal husbandry. Several studies claim that tail handling is aversive, while tunnel handling seems to have a lesser impact on animal welfare. However, most of these studies investigated the effect of handling performed in an unusually high frequency and prolonged duration, not matching laboratory routines. Therefore, the aim of our study was to investigate the impact of weekly cage change using tail versus tunnel handling on male and female C57BL/6J and CD-1 mice. Locomotion and exploratory activity as well as anxiety-related behaviour were measured. Moreover, the animals’ interest in social partners and social novelty as well as voluntary interaction with the handler were assessed. Reactivity and repeated activation of the HPA axis were monitored using corticosterone levels and adrenal gland and thymus weights. Only very few of the measured behavioural and stress physiological parameters differed significantly between the two handling groups, with varying direction. Our comprehensive analysis could thus reveal no consistent evidence supporting the superiority of one method over the other in terms of welfare of the handled mice.

**Supplementary Information:**

The online version contains supplementary material available at 10.1038/s41598-025-07384-w.

## Introduction

Handling methods for laboratory mice are an important and intensely debated topic in light of the refinement of animal husbandry and testing. Refinement, one of the 3Rs, focuses on improving conditions for animals in research by advancing experimental procedures and improving the housing environment^1,2^. Hence, limiting exposure to stressful events during routine handling is a critical aspect impacting animal welfare. Especially in laboratory mice, the most widely used vertebrate species in biological and biomedical research^3^, implementing refinement is crucial. Therefore, different handling methods for translocating mice to a novel cage or an experimental setup have been investigated in recent years. The most common handling method, i.e., handling mice by their tails, has become less popular due to concerns about a possible impact on animal welfare^4^. As a result, presumably less aversive methods, such as tunnel handling, have been advocated. Findings supporting the tunnel handling method have been published, showing that tunnel-handled mice showed more voluntary interaction with the handling device than tail-handled mice^4–13^. Additionally, an increase in exploratory behaviour and a decrease in anxiety-related behaviour were demonstrated at least in some studies^4,7–10,12,14^. Interestingly, these effects also seemed to persist when the animals were subjected to more invasive experimental procedures, such as injections or oral gavage^4,7,8,10^.

However, a noteworthy drawback of many of these studies is the applied frequency and duration of handling. Mostly, animals were handled in high frequencies (daily for nine subsequent days) and for long durations (e.g., 30 s) that do not reflect routine laboratory procedures. Fewer studies have applied handling frequencies and durations comparable to the everyday lab and animal care work or compared low and higher frequencies/durations, yielding inconsistent results^7,11,13,15–18^. For example, animals routinely handled by tail showed more agitation and were less easy to handle (by cup handling) following transportation compared to tunnel-handled mice^17^. In comparison to routine handling by tail using forceps, tunnel-handled C57BL6/J mice produced more pups and had fewer pre-weaning litter losses^15^, while no differences in breeding productivity were found in tail- and tunnel-handled BALB/cJ and CD-1-IGS mice^16^. Regardless of handling duration, females handled by tail interacted less with the experimenter in the voluntary interaction test than tunnel-handled mice^7^. Regarding behavioural test outcomes, one study found no handling-related alterations in anxiety-related behaviour in the Elevated Plus-Maze test (EPM) when handled on a routine basis (C57Bl/6J & BALB/cJ)^18^, while another found tail-handled females (C57Bl/6JOla/Hsd) spending less time on the open arms compared to tunnel-handled individuals^7^. In addition, routine handling in some cases led to strain-dependent differences, e.g., regarding locomotion in the Open Field test^18^ and anxiety-related behaviour in the EPM^13^ or sex and/or handling-frequency dependent changes in welfare-related parameters^11^.

Although the mentioned published studies mostly claim that tail handling is more stressful than tunnel handling, the inclusion of physiological markers of stress and anxiety induced by the different handling methods is largely missing. Therefore, evaluating hormonal stress responses towards either handling method would give important additional insights into possible impacts on animal welfare. The sparse evidence that is currently available shows several discrepancies^11,13,14,19^ and is thus not conclusive.

**Fig. 1:**
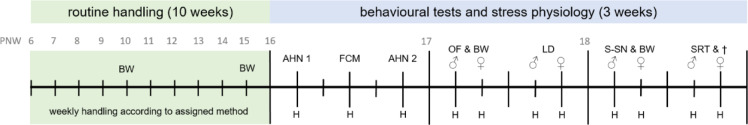
Experimental timeline. Starting at 6 weeks of age, animals were routinely handled once weekly for 10 weeks, either by the tail or tunnel handling method. Afterwards, behavioural and stress physiological assessments were performed for 3 weeks. Body weight was measured at 10, 15, 17 and 18 weeks of age. AHN, Approach to handler and novelty test, BW, body weight, FCM, faecal corticosterone metabolite analysis, LD, Light–Dark Box test, H, handling, OF, Open Field test, PNW, post-natal week, SRT, stress reactivity test assessing HPA axis reactivity, S-SN, Sociability and Social Novelty test.

Hence, in our study, in addition to a number of relevant behavioural tests investigating locomotion, exploration, anxiety-related and social behaviour, an in-depth analysis of physiological markers for acute and chronic stress responses was performed in order to investigate the impact of routine handling methods (tail vs. tunnel handling) on animal welfare of laboratory mice of both sexes. We selected two commonly used strains for our study, one inbred (C57BL/6J; B6) and outbred (CD-1), as it is known that different genetic backgrounds might show differences in behavioural and physiological reactions^20–24^. We hypothesised differential handling-related responses in exploration, anxiety-related and social behaviours as well as in stress responses in both strains and sexes. The experimental timeline and the used behavioural and stress physiological paradigms are outlined in Fig. 1.

## Results

The results presented in this section focus on handling-related effects and the interaction of handling with other factors. Apart from these effects, some sex differences were detected. As those are not the main focus of the study, these are presented in the corresponding Supplementary Material (Supplementary Tables S1–S8).

Handling time was measured during cage changes (time from opening the cage until the last mouse entered the new cage with all four paws). Overall, tunnel handling took longer than tail handling (about 40 s. vs. 10 s. for the two mice per cage). Handling times of the animals did not change much over the ten-week study period. Details are presented in Supplementary Table S9 and the Methods section.

### Behavioural tests

#### Open field test

**Fig. 2:**
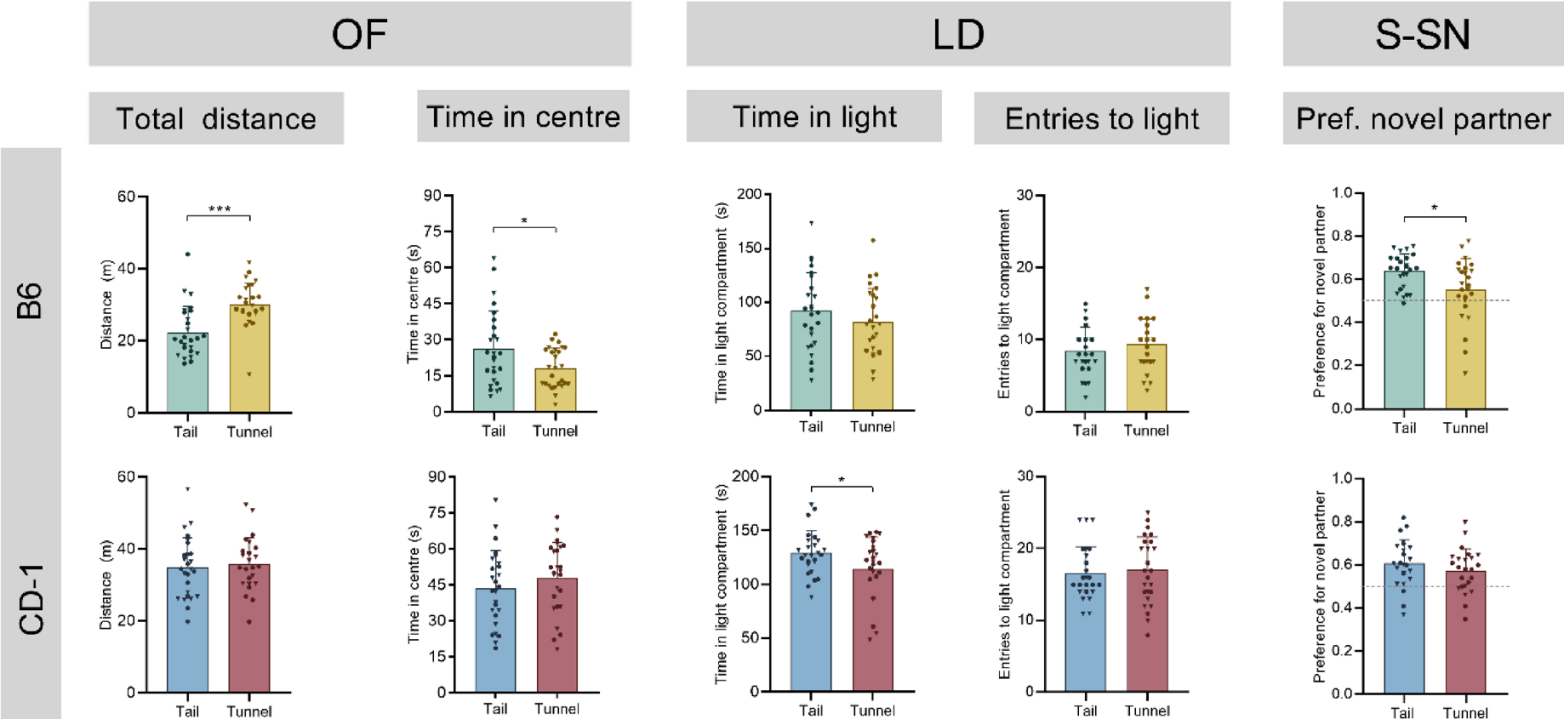
Results of the behavioural tests of C57BL/6J (B6; top panels), and CD-1 mice (bottom panels) either handled by tail or tunnel. Key results of the Open Field test (OF; distance travelled, time in the centre), the Light–Dark Box test (LD; time in and entries to the light compartment) and the Social Novelty phase of the Sociability and Social Novelty test (S-SN; preference for novel social partner) are presented. Data are depicted as means + SD and individual values for each mouse (males: circles, females: triangles). Statistics: two-way ANOVA with handling method and sex as fixed factors. Handling-related differences are presented in the graphs: *p < 0.05, ***p < 0.001; n = 26–27 per group.

The Open Field (OF) test was performed to assess locomotor activity and exploration. Overall, only a few statistically significant differences were observed between tail- and tunnel-handled mice (Fig. 2 and Supplementary Table S1). In C57BL/6J (B6) mice, tunnel-handled mice travelled a greater distance compared to tail-handled mice (main effect of handling: F_1,49_ = 17.735, *p* < 0.001). In addition, tail-handled B6 mice spent more time in the centre of the OF compared to their tunnel-handled counterparts (main effect of handling: F_1,49_ = 5.114, *p* = 0.028). In CD-1 mice, no handling-related differences were found in any of the analysed parameters of the OF test (Fig. 2 and Supplementary Table S1).

#### Light–dark box test

The Light–Dark Box (LD) test was used to investigate anxiety-related behaviour of mice handled by tail or tunnel. In B6 mice, no significant effect of handling method was observed in any of the analysed parameters (Fig. 2 and Supplementary Table S2). In CD-1 mice, tail-handled animals spent significantly more time in the light compartment than tunnel-handled individuals (main effect of handling: F_1,48_ = 4.629, *p* = 0.036; Fig. 2 and Supplementary Table S2).

#### Sociability and social novelty test

The Sociability and Social Novelty (S-SN) test was performed to investigate the interest of the mice in novel social stimuli. During the *Sociability phase*, it was tested whether the animals preferred an unfamiliar social partner (unfamiliar male of the same strain presented under a wire mesh cup) over an unfamiliar object (wire mesh cup without a social stimulus animal). Regarding handling-related effects, in B6 mice no differences between tail- and tunnel-handled mice were detected in the assessed parameters (Supplementary Table S3) and in CD-1 mice, only the distance travelled was affected (F_1,48_ = 8.512, *p* = 0.005), revealing a greater distance travelled in tunnel- compared to tail-handled mice. Test statistics of all other analysed parameters are presented in Supplementary Table S3.

In the *Social Novelty phase* it was tested whether the mice preferred a novel social stimulus (unfamiliar mouse of the same strain placed under the previously empty wire mesh cup) over a familiar one. Most parameters analysed did not show a handling related difference (Supplementary Table S4). However, tail-handled B6 mice showed a significantly higher preference for the novel compared to the familiar social partner (main effect of handling: F_1,47_ = 7.073, *p* = 0.011; Fig. 2) in comparison to tunnel-handled B6 mice. Such a difference was not seen in CD-1 mice (F_1,45_ = 1.499, *p* = 0.227; Fig. 2 and Supplementary Table S4).

#### Approach to handler and novelty (AHN) test

**Fig. 3:**
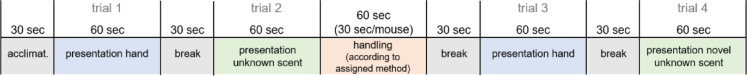
Schematic overview of the Approach to Handler and Novelty (AHN) test. The test was performed in the home cage of the mice (two animals housed per cage). Before starting the test, the cage lid and nesting material were removed (acclimatisation). The AHN test consisted of four trials, two before and two after handling the mice with the assigned method. The AHN test was performed twice with a break of three days between the two test sessions.

The AHN test was performed twice after 10 weeks of handling (see experimental timelines in Figs. 1 and 3) in the home cage of the animals. The direct interaction of the mouse with the gloved hand of the experimenter was interpreted to reflect interest or avoidance of the experimenter, while interaction with an unfamiliar scent (food-related baking aromas), reflected interest or avoidance of a novel handling-unrelated stimulus. In this test, direct investigation was defined as the nose of the mouse being within one head-length of the experimenter’s hand or the cotton swab presenting the scent. The animals were allowed to investigate the hand of the experimenter and subsequently an unknown scent (trial 1 & 2). Afterwards, both animals of the cage were handled for 30 s each according to their assigned handling method, followed by another presentation of the experimenters’ hand and a new, unfamiliar scent (trial 3 & 4). We expected that animals would avoid the hand, if it was perceived as aversive and that this effect would be stronger directly after a handling event. As the pair-housed animals were tested simultaneous in their home cage, the data was averaged by cage.

A linear mixed model analysis was applied, with handling method, sex, trial (before/after handling) and stimulus (hand/scent) as fixed factors and cage and session as random factors.

**Fig. 4:**
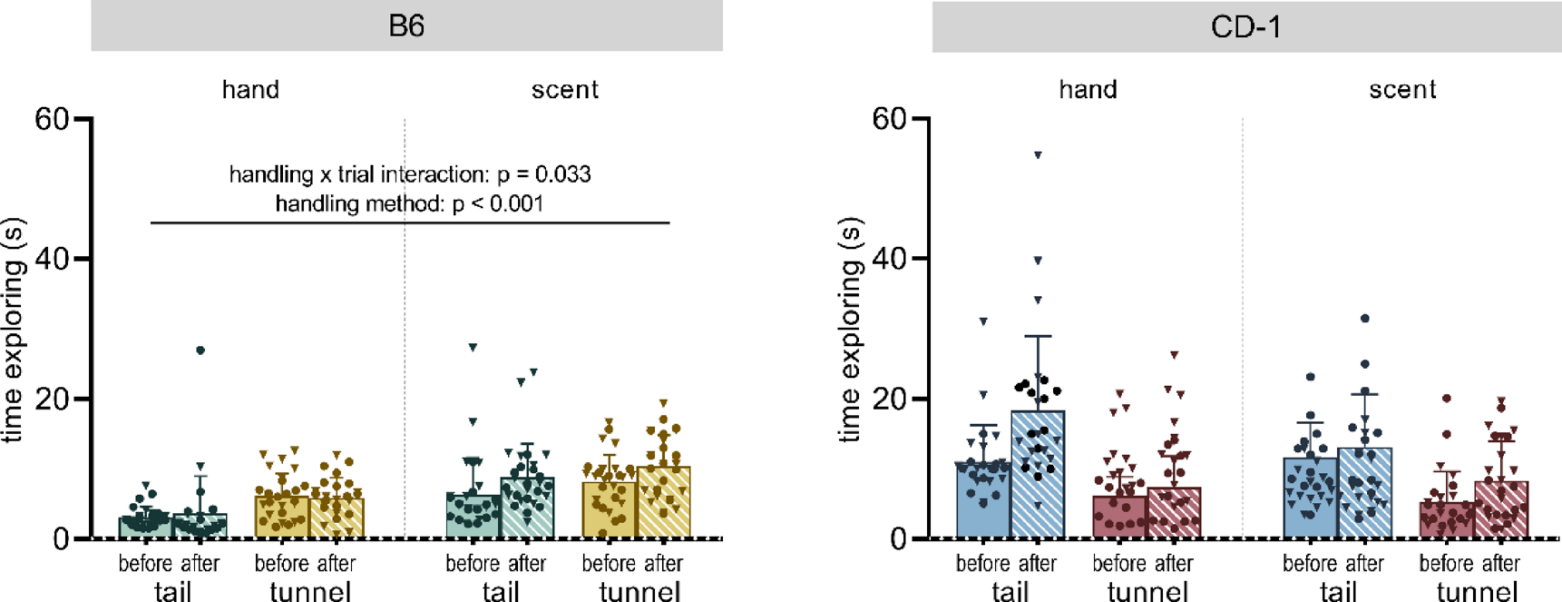
Time spent exploring the hand or scent during the Approach to Handler and Novelty (AHN) test of C57BL/6J (B6) (left panel) and CD-1 mice (right panel). The hand and the scent were presented for 60 s before and after handling the mice using the assigned handling method (tail or tunnel). Data are depicted as means + SD and individual values (please note that there are two values per cage representing the two testing sessions; males: circles, females: triangles). Statistics: linear mixed model with handling method, sex, trial (before/after) and stimulus (hand or scent) as fixed factors and cage and session as random factors. Handling-related differences are presented in the graphs, all statistical comparisons are shown in Table 1; n = 13–14 cages per group.

In B6 mice, a handling method x trial interaction (F_1,189_ = 4.618, *p* = 0.033) was found (Fig. 4 and Table 1). Post hoc t-tests showed that in comparison to tail-handled mice, tunnel-handled mice spent more time investigating the stimulus in all trials (before handling: t = 3.536, *p* < 0.05; after handling: t = 5.687, *p* < 0.05 for both comparisons after Bonferroni-Holm correction). Moreover, tunnel-handled mice investigated the presented stimuli more after being handled than before being handled (t = 2.891; *p* < 0.05 after Bonferroni-Holm correction). Additionally, there was a significant main effect of handling method (F_1,27_ = 32.508, *p* < 0.001), as tunnel-handled B6 mice spent more time investigating the stimuli than tail-handled mice, while B6 mice generally investigated the novel scent more than the experimenter’s hand (F_1,189_ = 17.197, *p* < 0.001). Moreover, a main effect of trial (F_1,189_ = 5.483, *p* = 0.020) was detected.

**Table 1:** Test statistics for the linear mixed model ANOVA for the Approach to Handler and Novelty (AHN) test of C57BL6/J (B6) and CD-1 mice with handling method (tail or tunnel), sex (male/female), trial (before/after handling) and stimulus (experimenters’ hand or novel scent) as fixed factors and cage (means of both animals in the cage) and session as random factors.

Approach to handler and novelty test		
ANOVA	Time spent investigating stimulus (hand or scent)	
Fixed factors	B6	CD-1
Handling method	F_1,27_ = 32.508 ***p* < 0.001**	F_1,25.489_ = 0.526 *p* = 0.475
Sex	F_1,27_ = 1.693 *p* = 0.204	F_1,25.489_ = 0.934 *p* = 0.343
Trial (before/after handling)	F_1,189_ = 5.483 ***p* = 0.020**	F_1,179.789_ = 23.141 ***p* < 0.001**
Stimulus (hand or scent)	F_1,189_ = 17.197 ***p* < 0.001**	F_1,179.789_ = 93.739 ***p* < 0.001**
Handling method x sex	F_1,27_ = 1.876 *p* = 0.182	F_1,25.489_ = 0.471 *p* = 0.499
Handling method x trial	F_1,189_ = 4.618 ***p* = 0.033**	F_1,179.781_ = 1.748 *p* = 0.188
Handling method x stimulus	F_1,189_ = 0.632 *p* = 0.428	F_1,179.781_ = 3.048 *p* = 0.083
Sex x trial	F_1,189_ = 5.055 ***p* = 0.026**	F_1,179.781_ = 0.217 *p* = 0.642
Sex x stimulus	F_1,189_ = 1.082 *p* = 0.300	F_1,179.781_ = 0.085 *p* = 0.771
Trial x stimulus	F_1,189_ = 0.274 *p* = 0.601	F_1,179.781_ = 2.765 *p* = 0.098

Statistically significant results are marked in bold.

In CD-1 mice, no significant effects of handling method on investigating the hand or scent were detected (Fig. 4 and Table 1). There was a main effect of trial, showing that the investigation was longer after being handled than before being handled (F_1,179.8_ = 23.141, *p* < 0.001) and a main effect of the presented stimulus, showing that CD-1 mice explored the hand of the experimenter longer than the scent (F_1,179.8_ = 93.739, *p* < 0.001). Statistical details can be found in Table 1.

### Physiological measures

#### Body weight

Body weight was assessed at four time points across the 10-week handling period and the three behavioural testing weeks (data shown in Supplementary Table S5).

In B6 mice, a repeated-measures ANOVA revealed a significant age x handling x sex interaction (F_3,147_ = 3.510, *p* = 0.017). Post hoc t-tests showed no handling-related differences at the different measuring time points (*p* > 0.05 for each time point after Bonferroni-Holm correction), but, as expected, male mice were significantly heavier than female mice (*p* < 0.05 for each time point after Bonferroni-Holm correction).

In CD-1 mice, no handling related differences in body weight were detected (age x handling method: F_3,144_ = 0.295, *p* = 0.829, Supplementary Table S5).

#### Faecal glucocorticoid metabolites as a marker of stress in response to handling

**Fig. 5:**
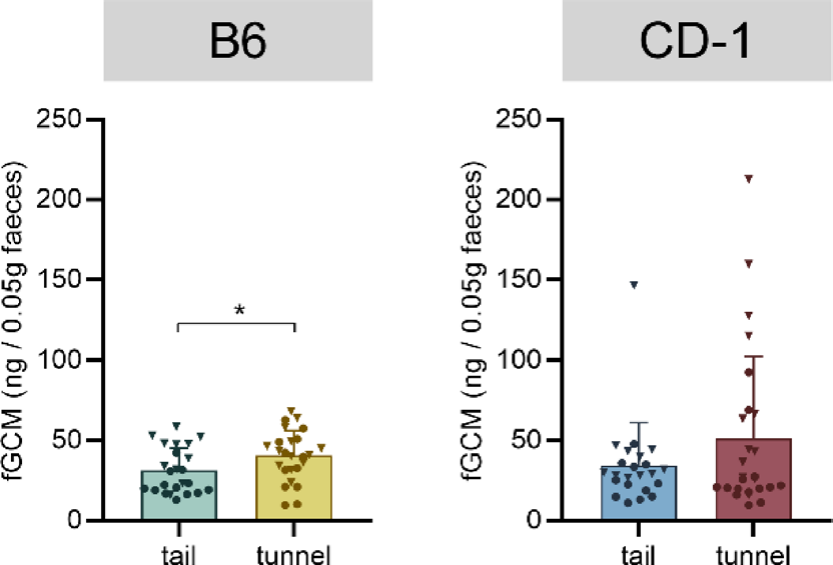
Faecal glucocorticoid metabolite (fGCM) concentrations (ng/0.05 g faeces) as a physiological stress marker in response to handling by tail or tunnel in C57BL/6J (B6; left panel) and CD-1 (right panel) mice. Data are presented as means + SD and individual values (males: circles, females: triangles. Statistics: two-way ANOVA with handling method and sex as fixed factors. Handling-related differences are presented in the graphs: *p < 0.05; n = 23–26 per group.

In B6 mice, following handling, there was a significant main effect of handling method (F_1,45_ = 4.894, *p* = 0.032) on faecal glucocorticoid metabolite (fGCM) concentrations. Tunnel-handled mice showed higher fGCM levels than tail-handled B6 mice (Fig. 5 and Supplementary Table S6). In CD-1 mice, no statistically significant handling-related differences were observed (F_1,45_ = 2.931, *p* = 0.094, Supplementary Table S6).

#### HPA axis reactivity in response to a psychological stressor

**Fig. 6:**
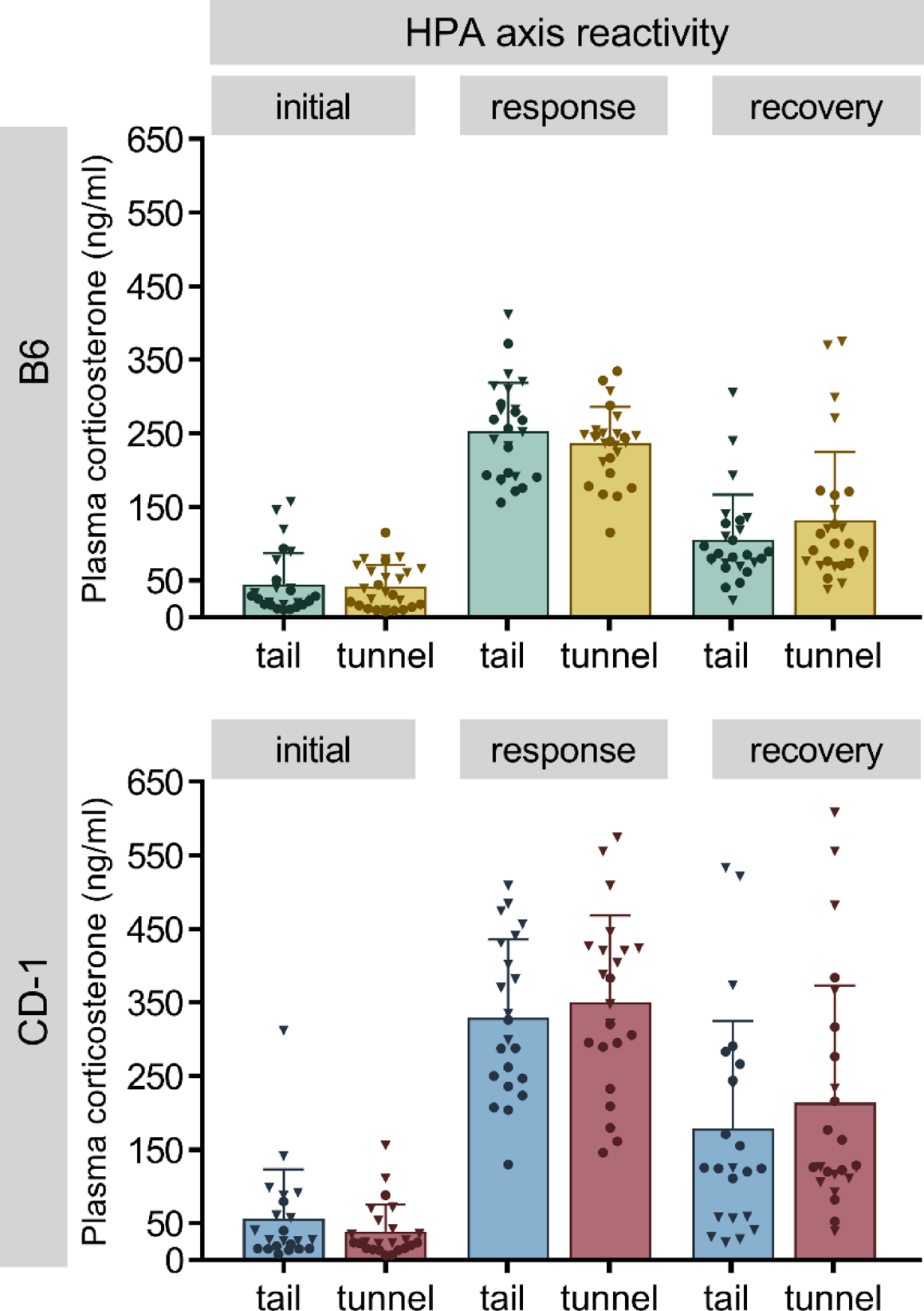
Plasma corticosterone concentrations (ng/ml) of C57BL/6J (B6; top panel) and CD-1 mice (bottom panel). Corticosterone levels are shown at baseline (initial), after a 15-min restraint (response) and after a recovery period of 75 min after stressor termination (recovery). Data are depicted as means + SD and individual values (males: circles, females: triangles). Statistics: repeated-measures ANOVA with handling method and sex as between-subject factors. No significant handling-related differences were detected; n = 20–27 per group.

To investigate HPA axis reactivity, plasma corticosterone concentrations were measured at baseline (initial), after a 15-min restraint (response) and after a recovery period of 75 min after stressor termination (recovery). Overall, in both mouse strains, no handling related differences were detected by repeated-measures ANOVA (Fig. 6 and Supplementary Table S7).

#### Organ weight as a chronic stress marker

**Fig. 7:**
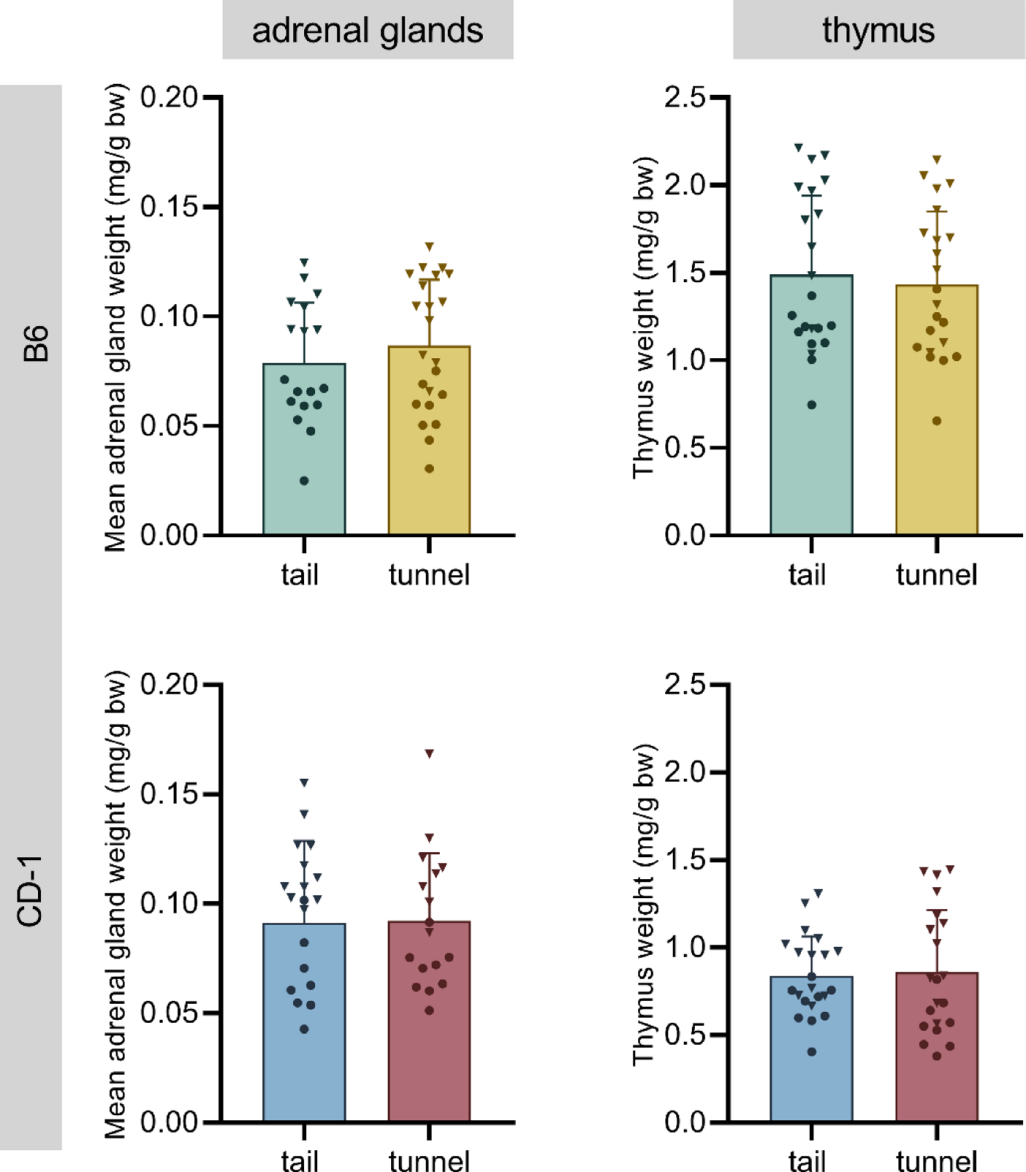
Organ weight relative to body weight (bw) of adrenal glands (mean of the left and right adrenal gland) and thymus of C57BL/6J (B6; top panels) and CD-1 mice (bottom panels). Data are depicted as means + SD and individual values for each mouse (males: circles, females: triangles). Statistics: two-way ANOVA with handling method and sex as fixed factors. No significant handling-related differences were found; n = 21–22 per group.

In B6 and CD-1 mice, the weight of the adrenal glands (mean of left and right adrenal) and the thymus, both relative to body weight, were not significantly affected by handling method (Fig. 7), although the expected sex differences were found (heavier in females, Supplementary Table S8).

## Discussion

As handling is one of the laboratory routine procedures that all laboratory mice experience regularly, it is important to minimise potential negative impacts on animal welfare. Therefore, our study aimed to investigate whether handling mice by tail or tunnel, at a frequency and duration usually applied during routine housing and testing in biomedical research, affects the animals differently regarding behavioural and physiological markers of animal welfare.

Overall, for both mouse strains we found very few statistically significant and rather inconsistent handling-related effects on the measured behavioural and physiological parameters. Exploratory behaviour, locomotion and anxiety-related behaviour showed little to no handling-related alterations. In our OF test (five minutes test duration), B6 tunnel-handled mice travelled a greater distance than tail-handled individuals, while tail-handled individuals spent more time in the centre of the arena. A previous study^18^ also applying less frequent handling (brief handling twice a week for cage changes) found no significant difference in locomotor activity in the OF test (60 min test duration) between female tail- and tunnel-handled B6 mice, but observed significant effects for BALB/c mice. Similarly, the locomotor activity was slightly increased in tunnel-handle CD-1 mice in the sociability phase of our S-SN test. It seems possible that letting the animals voluntarily enter the arena (emerging from the tunnel) versus placing them down by their tail impacts the initial locomotor activity in the behavioural tests, which could at least partially account for the observed differences. Studies applying higher handling frequencies or durations also found similar effects regarding locomotion in the OF, yet contrasting results for the time spent in the centre of the arena^7,8^ that were still observed 3 weeks after the offset of daily handling^9,12^. While this might be explained by handling frequency, other factors such as illumination intensity of the arena, location of placement of the mouse, sex and strain are important factors as well and cannot be excluded.

Anxiety-related behaviour was measured in the Light–Dark Box (LD) test. We found no significant differences between the handling groups in any relevant parameter in B6 mice. These findings are in line with another recent study that handled mice twice a week, where B6 mice also did not show handling-related effects in the Elevated-Plus Maze (EPM) test^13^. Yet, the tail-handled CD-1 mice in our study spent more time in the light compartment compared to their tunnel-handled counterparts, indicating less anxiety-related behaviour. However, the other parameters tested in the LD test were not significantly altered by handling method. Contrasting to our results, in a study by Gouveia and Hurst, anxiety-related behaviour was shown to be increased in tail-handled mice compared to tunnel-handled individuals, independent of handling frequency^7^. This might be explained by a different strain being used (BALB/cOlaHsd), mice being tested during the dark phase and probably most importantly, by placement into the EPM facing the open arm vs facing the closed arm^7^. Studies investigating a longer duration and higher frequency of handling on anxiety-related behaviour, mainly found that tail-handled individuals showed increased anxiety-related behaviour in the LD^14^ and EPM test (e.g.,^4,7,12^) or produced inconclusive results^8^.

In addition to exploration and anxiety-related behaviours, social behaviour was assessed in the Sociability and Social Novelty test. While general interest in an unfamiliar social partner (unfamiliar male mouse of the same strain) measured in the *Sociability Phase* was not significantly affected by handling method, tail-handled B6 mice showed a greater preference for a novel conspecific in the *Social Novelty Phase* than their tunnel-handled counterparts, indicating a stronger interest in social novelty, which is expected for a socially living species^25^. Overall, social behaviour, as measured in this test, was not strongly altered by handling method.

In the Approach to Handler and Novelty (AHN) test, tunnel-handled B6 mice spent more time investigating the presented stimuli (hand or scent) than tail-handled mice and they also increased their exploration after being handled. This is in line with results found in other studies (e.g.,^4–13^). It seems that tail-handled B6 mice might perceive the hand as more of a threat compared to tunnel handled mice. In contrast, CD-1 mice were rather unaffected by handling method in the AHN test. Animals of both handling groups and strains investigated the stimulus more after being handled than before, indicating a possible activating effect of being handled. The introduction of a handling-unrelated stimulus (an unfamiliar baking aroma on a cotton swab) to the test in our study was a new approach for disentangling the possible avoidance of the handler and the avoidance of general novelty introduced to the cage. Interestingly, independent of handling method, B6 mice spent more time investigating the scent compared to the hand, while this was the reverse in CD-1 mice. It might therefore be speculated, that B6 mice in general are more anxious towards handling-related than handling-unrelated stimuli. For interpreting the results, it has to be kept in mind that in studies by other researchers usually the handling device (i.e., the hand or the tunnel) is presented, and the time investigating either hand or tunnel is evaluated (e.g.,^4^). We changed this protocol as we think that the tunnel, in general, is likely to be more attractive to the animals, as it provides shelter and more space for exploration than the experimenter’s hand. This argument is supported by findings from a study presenting the tunnel and the hand, irrespective of handling method, where the tunnel was explored more than the hand^13^. Thus, the previously published differences between tail- and tunnel-handled animals might have simply been due to a general difference in interest between hand and tunnel. Yet, similar to our approach, a study by Henderson and colleagues^10^ also only investigated the interaction with the hand. Here, animals handled by tunnel spent more time exploring the hand compared to tail-handled individuals on days five and nine of daily handling but not on day one^10^. However, the mouse strain investigated in that study was different (BALB/c) and handling duration and frequency were higher^10^. Another study that handled CD-1 mice on a routine basis using cup handling investigated the effects of a tail- or non-tail restraint technique in a similar design also only presenting the experimenter’s hand before and after restraint and found no restraint-related difference in voluntary interaction with the handler^17^. A difference that has to be kept in mind is the timing of our AHN and the voluntary interaction test (VIT) in previous studies (see citations above). In our study, animals were accustomed to handling for several weeks, while in the cited studies, the VIT was mostly performed several times, starting with the onset of applying the different handling methods. Therefore, in light of the different approaches, direct comparison and interpretation of the results is difficult. Overall it seems that in our study, tail-handled B6 mice show a more cautious response towards the handler, while CD-1 mice do not show this differential response. However, in general, over-interpretation of these findings should be avoided. Unlike the classical behavioural tests (OF, LD, EPM, S-SN etc.), the validity of the VIT and AHN has not been proven yet, for instance by showing a rescue of the reduced time exploring the experimenter’s hand after anxiolytic drug treatment in tail-handled animals.

Some aspects that need to be kept in mind when interpreting the data presented in our study is that in contrast to many studies investigating tail and tunnel handling, the mice did not have the tunnel in their home cage, but it was stored in the food hopper. One study showed however, that once B6 mice were experienced in tunnel handling, the familiarity with the tunnel (shared tunnel between cages vs. home cage tunnel) did not significantly affect the interaction with the handling device and did not lead to differences in anxiety-related behaviour in the EPM test^5^. Moreover, in our study handling time was longer for tunnel- compared to tail-handled mice (for details see Method section and Table S9), which might have had an impact on the behavioural and physiological responses investigated. Furthermore, all tests were performed during the light-phase, which was different in other studies.

In general, in our study the outcomes of the classical behavioural tests (OF, LD and S-SN) showed only a few effects of the different handling methods and the direction of these effects were not consistent (tunnel-handled mice showed more locomotion, while tail-handled mice showed less anxiety-related behaviour and more interest in a novel social stimulus). In addition, the effects were often only found in one of the two strains, indicating no generalizable and/or strong effects. In the AHN, tail-handled B6 mice showed a more consistent avoidance response towards the handler, while we found no significant handling-related differences for CD-1 mice.

Regarding the physiological parameters involved in the stress response and with relevance for assessing animal welfare, data from previous studies are scarce and do not provide clear results. For example, a study by Ono and colleagues^19^ could show that plasma corticosterone levels were higher in tail-handled C57BL/6 compared to the control group, yet in BALB/c mice, tunnel-handled animals had higher levels than tail-handled individuals. In our study, we did not find handling-related effects in plasma corticosterone levels (neither at baseline, nor after a psychological stressor), which is also in line with a recent study that did not find differences in plasma corticosterone^13^. However, our fGCM analysis revealed that tunnel-handled B6 mice showed a slightly but significantly stronger activation of the HPA axis in response to handling. This is divergent to other studies showing no difference due to handling method in faecal corticosterone metabolites^11,14^. However, in these studies, HPA axis activation was not measured in a manner directly related to the handling event ^11,14^.

In addition, the weight of the adrenal glands and thymus, two organs that are involved in the stress response and represent reliable markers for chronic stress (e.g.,^26^), did not show any significant differences. While this is in line with previous findings^13^, others found an increase in adrenal gland weight following tail handling^12^. This difference is likely to be explained by the higher frequency and duration of handling (once or twice a week vs. daily handling on nine consecutive days). Thus, from the comprehensive data collected in our study, no evidence for a conclusive effect of the handling method on physiological stress markers was found.

Taken together, our extensive and detailed analysis did not reveal clear and consistent differences in behavioural patterns between mice handled by tail or by a tunnel that was not present in the home cage. Moreover, we did not observe a strong impact of the handling method on stress physiological parameters in B6 and CD-1 mice. Overall, while there are a few statistically significant differences between handling groups, these were only observed in some of the analysed parameters and no consistent pattern favouring one handling method over the other became apparent. We cannot draw direct conclusions about strain differences, as strains were analysed separately, but there were slightly more handling-related differences between tail- and tunnel-handled B6 than between tail- and tunnel handled CD-1 mice. Thus, based on our comprehensive evaluation, tail- and tunnel-handling seem to have no consistent differential impact on behavioural and physiological markers of welfare when applied on a routine basis.

### Animals, materials and methods

The presented work complies with current regulations covering animal experimentation in Germany and the European Union (European Directive 2010/63/EU). The authors also complied with the ARRIVE guidelines. All experiments were announced to and approved by the Lower Saxony State Office for Consumer Protection and Food Safety (LAVES, licence no. 22/00177) and the ‘Animal Welfare Officer’ of Osnabrück University.

### Animals

Male and female mice of the C57BL/6J (B6) and CD-1 strains were obtained from our in-house breeding colonies. In total, n = 112 animals were tested (n = 14 per group; deviations from this sample size were due to technical reasons in the different tests. Details can be found in the Supplementary Material). For estimation of sample size, an a priori power analysis was conducted based on results from previous studies to ensure the detection of biologically relevant differences between handling methods with a power of 0.8 (β = 0.20, α = 0.05 and effect size (f) = 0.40). Animals were housed in open Macrolon Type II long cages (365 × 205 x 140 mm (l x w x h)) with bedding (Aspen wood chips, 4HK, Altromin GmbH, Lage, Germany) and nesting material (wood shavings (NBF E-011, Altromin GmbH, Lage, Germany)). Food (hybrid pellets (No 1328, Altromin GmbH, Lage, Germany)) and water were provided ad libitum. The animal housing rooms were kept at a 12:12 h light–dark cycle (lights on at 8 am) with a constant room temperature of 22 ± 2 °C and relative humidity of 55 ± 10%. All tests were performed in the first three hours of the light phase, unless stated otherwise.

From weaning (at four weeks of age) until the start of the experiment (at six weeks of age), the animals were handled by tail-handling. Starting at six weeks of age, mice were kept in same-sex groups of two individuals. Half of the animals were handled by tail, and the other half were handled by tunnel handling (details see below). Within each cage, the same handling method was applied, and the handling method was assigned in a random fashion, yet ensuring that all groups consisted of the same number of animals. Cages of tail- and tunnel-handled animals were placed alternately in the racks. The handling tunnel was stored in the food hopper of the respective cage lid and was not provided as an enrichment item inside the cage, as we wanted to focus on effects of handling independent from the tunnel as cage enrichment.

Starting from postnatal week (PNW) six, animals were handled once a week for 10 weeks for cage change and health inspection according to their assigned handling method. Alternating between weeks, mice of either the tail handling or the tunnel handling group were handled first. Body weight was assessed at the age of 10, 15, 17 and 18 weeks (see experimental timeline in Fig. 1).

### Handling methods

Four experimenters, equally experienced with both handling methods, performed the handling. For tail handling, mice were gently picked up at the middle of their tails, placed on the back of the gloved experimenter’s hand (gloves: Peha-soft nitrile, Paul Hartmann AG, Heidenheim, Germany), and transferred to a new cage or test apparatus for behavioural testing. Here, they were lifted by the tail again and placed down gently.

For tunnel handling, the tunnel (red translucent tunnel; Ø 5 cm, length 10 cm, Plexx BV, The Netherlands) was placed on the bedding in the cage (while in the hand of the experimenter). The mice were allowed to enter the tunnel voluntarily. If they did not enter the tunnel on their own within a couple of seconds, they were gently guided into the tunnel by the free hand of the experimenter. Once the animal was inside the tunnel, the open ends were loosely covered with both gloved hands to ensure that the mouse stayed in the tunnel, and the mouse was transferred to a new cage or the test apparatus for behavioural testing. Here, the tunnel was tilted slightly backwards and the mouse could voluntarily leave the tunnel.

Handling time was also taken into account and was measured during cage changes (time from opening the cage until the last mouse entered the new cage with all four paws). Overall, tunnel handling took longer than tail handling (about 10 s. vs. 40 s.), and the time it took to handle the animals did not change much over the ten-week study period (Supplementary Table S9). This is somewhat different from other studies applying both handling methods and not reporting this considerably longer handling times (tunnel handling either took only a couple of seconds longer^27^ or showed a 1.7-fold difference^28^). This might be explained by the fact that unlike in our study, the animals in those studies had access to the tunnel in their home cage and thus were more familiar with the handling device^27^. Moreover, the longer time for cage change using tunnel-handling in our study might be due to the period each animal was allowed to enter the tunnel voluntarily. In cases, when an animal did not enter the tunnel even when gently guided by the experimenter, the experimenter stepped back from the cage for several seconds, leaving the tunnel in the cage, giving the animals more time and then started the handling procedure again in an attempt to minimise handling-stress, thus expanding the total time for the cage transfer of these mice.

### Behavioural measures

#### Open field test

The Open Field test (OF) was performed to measure differences in locomotor activity and explorative behaviour. Each mouse was placed in the outer part close to the wall of the test apparatus facing away from the centre of the circular arena (Ø 600 mm, wall height 400 mm, illumination about 15 lx) using their assigned handling method. The OF test lasted for five minutes, and afterwards, the animals were placed back in their home using their assigned handling method. The OF test was recorded and analysed using the tracking software ANYmaze (version 7.0, Stoelting Europe). The parameters analysed were the total distance travelled, the time spent in the centre (inner 300 mm of the OF arena) and the number of entries to the centre. Both mice of the same cage were tested simultaneously in identical apparatuses. The different handling groups were tested in a counterbalanced design. Males and females were tested on consecutive days. For obvious reasons, the experimenters were not blind to the handling method and strain while conducting the tests. However, the automated tracking software ensured unbiased behavioural analyses.

#### Light–dark box test

The Light–Dark Box (LD) test was performed to assess anxiety-related behaviour. The apparatus consisted of a dark compartment (illumination < 15 lx; 185 × 200 mm (l x w)) and a brightly lit compartment (illumination 700 lx; 290 × 200 mm (l x w)) that were connected by a short tunnel (45 × 60 mm (l x w)). The mice were placed in the dark compartment, facing away from the tunnel, using their assigned handling method, and they had five minutes to explore the LD apparatus. Afterwards, the mice were returned to their home cage using their assigned handling method. The LD test was recorded and analysed using the tracking software ANYmaze (version 7.0, Stoelting Europe). The parameters analysed were the distance travelled in the light compartment as well as the number of entries and the time spent there. Both mice of the same cage were tested simultaneously in identical apparatuses. The different handling groups were tested in a counterbalanced design. Males and females were tested on consecutive days. For obvious reasons, the experimenters were not blind to the handling method and strain while conducting the tests. However, the automated tracking software ensured unbiased behavioural analyses.

#### Sociability and social novelty test

The Sociability and Social Novelty test (S-SN) was performed to investigate general social behaviour and interest in social novelty. This test consisted of two phases. In the ‘*Sociability Phase*’ (SP), the interest in an unfamiliar male mouse of the same strain under a wire mesh cup compared to an empty wire mesh cup was investigated. During the second phase, the ‘*Social Novelty Phase*’ (SNP), a second unfamiliar conspecific was presented under the previously empty cup in addition to the now familiar conspecific. Both phases lasted 10 min each and were performed back to back. For detailed test description see below.

The S-SN apparatus (475 × 250 × 180 mm (l x w x h) could be divided into three compartments by sliding doors. Before the start of the sociability phase, the test mice were habituated to the test apparatus by being placed in the middle compartment for two minutes. Immediately afterwards, the sliding doors were removed, the sociability phase started, and the mice could freely explore the entire S-SN arena for 10 min. A wire mesh cup (Ikea pen holder, 100 × 100 × 110 mm (l x w x h)) was placed upside down at each of the far ends of the testing apparatus, touching the wall on one side. During the sociability phase, an unfamiliar male of the same strain was placed under one wire mesh cup, while the other cup remained empty and served as a novel object. After the SP, the sliding doors were re-introduced and the test mouse was gently guided to and confined in the middle compartment, before a second conspecific (another unfamiliar adult male of the same strain) was placed under the previously empty wire mesh cup. The sliding doors were then removed again for the start of the SNP. Both mice of the same cage were tested simultaneously in identical apparatuses. The location of the empty wire mesh cup was balanced across handling groups, mouse strains and sexes and the locations remained the same during the SP and SNP. Both mice of the same cage were tested simultaneously in identical apparatuses. The different handling groups were tested in a counterbalanced design. Males and females were tested on consecutive days. For obvious reasons, the experimenters were not blind to the handling method and strain while conducting the tests. However, the automated tracking software ensured unbiased behavioural analyses.

The S-SN test was recorded and analysed using the tracking software ANYmaze (version 7.0, Stoelting Europe). Parameters analysed were total distance travelled, entries to and time spent in the interaction zones (a 1 cm wide area around the three accessible sides of each wire mesh cup) as well as the preference ratio for the time as well as entries to the interaction zones (e.g.: SP: time at social partner/(time at social partner + time at object); NSP: time at novel social partner/(time at novel social partner + time familiar partner)). A ratio higher than 0.5 indicates a preference for the social partner over the object (SP) or the novel social partner over the familiar social partner (SNP).

#### Approach to handler and novelty test

The *Approach to Handler and Novelty* (AHN) test was performed after 10 weeks of handling and was loosely adapted from the *voluntary interaction test* (VIT) originally described by Hurst and West^4^. In contrast to the original protocol, both handling groups were allowed to investigate the hand of the experimenter (instead of the handling device assigned to their respective handling method (tunnel or hand)). In addition to the hand of the experimenter, the animals received the opportunity to investigate a stimulus unrelated to the handling procedure (novel scent). This was done to investigate if a lesser interest in the experimenter’s hand was due to aversive experiences during handling or a sign of general anxiety towards stimuli presented in the home cage.

Both animals from the same cage were tested simultaneously and data was averaged per cage. In total, two sessions of the AHN were performed, each consisting of four trials (timeline see Fig. 3). The two sessions were performed with a break of four days in between (see experimental timeline in Fig. 1). Briefly, the lid of the home cage and the nesting material were removed. After an acclimatisation period of 30 s, the gloved hand of the experimenter was held in the front left part of the cage, touching the bedding lightly with the fingertips. For 60 s, the mice could freely explore the hand (trial 1). After a 30-s break, an unknown scent (details see below) was presented on a cotton swab (cotton tip: 12 mm in diameter and 35 mm in length, wooden stick: 200 mm) in the same position as the hand was presented before (trial 2). The experimenter held the cotton swab (making sure the hand was not inside or above the cage), hovering about 1 cm over the bedding. Following these two trials, each mouse in the cage was handled for 30 s according to the assigned handling method. This involved either holding the mouse in the tunnel with the openings loosely covered by the experimenters’ hands or keeping the mouse on the hand while holding the animal by the tail. This was done in an attempt to rather closely follow previously published protocols, e.g., the Voluntary Interaction Test (e.g.,^4^). After handling, the mice again had the opportunity to explore the hand (trial 3) and another unfamiliar scent (trial 4) for 60 s each.

The scents used were baking aromas (LorAnn Oils, Lansing, Michigan, USA) diluted at a ratio of 1:100. It was previously established that “banana”, “cinnamon”, “raspberry”, and “lime” elicited a similar exploration in mice^29^. The order in which the scents were presented was balanced across handling group, strain, sex, session and trial. All animals were habituated to the cotton swab three days prior to the AHN test, where a cotton swap moistened with water was presented twice for 60 s (with a break of 30 s between trials) in the home cage, as described above.

The different handling groups were tested in a counterbalanced design. Males and females were tested on consecutive days. For obvious reasons, the experimenters were not blind to the handling method and strain while conducting the tests. However, videos were recorded of each trial of the AHN test, and the behaviour was analysed by experienced observers (Observer XT 16, Noldus, The Netherlands), who were blind to the handling method, the sex of the animals and the trial number (before/after handling). The direct interaction with the handler or the scent was scored and defined as follows: the head of the mouse is within one head length of the hand or the cotton swab. The interaction ends if the head is turned more than 90° away from either the hand or the cotton swab.

### Physiological measures

#### Stress response to handling

To investigate the endocrine stress response to handling, at 16 weeks of age, mice were handled for 30 s according to their assigned handling method and then returned to their home cage. Eight hours later, the mice were placed individually into Macrolon Type II cages (equipped with bedding, food and water) for one hour and then returned to their home cages. All faecal boli voided during this hour were collected and frozen at − 20 °C until further processing. The eight-hour interval was chosen because of the lag time between secretion of corticosterone into the blood and excretion of corticosterone metabolites in the faeces^30,31^. The collected faecal samples were analysed for immunoreactive corticosterone metabolites using a 5α-pregnane-3β,11β,21-triol-20-one EIA. Details regarding the development, biochemical characteristics and physiological validation of this assay are described by Touma and colleagues^30,31^. Moreover, the utilised EIA has proven well-suited to detect even small changes in adrenocortical activity in mice^31–34^. The processing of the samples before EIA analysis and a detailed description of the assay protocol have been published elsewhere^30^. The different handling groups were tested in a counterbalanced design. Males and females were tested on consecutive days.

#### HPA axis reactivity test

To investigate if the handling methods differentially affected the reactivity of the HPA axis to a standardised psychological stressor, a blood sample (initial value) was taken from the tail vessel^34^, after which the mouse was quickly led into a tube with air holes (50 ml falcon tube) in which they stayed for 15 min (restraint). While the mice were not physically immobilized, they could not escape the situation, inducing psychological stress^35^. For the collection of the initial sample, animals were removed from their cage by their assigned handling method and placed on the metal grid of a clean cage lid, where they were gently held by their tail (for details on this minimally invasive blood sampling technique see^34,35^).

After the 15-min restraint, a second blood sample (reaction value) was taken from the tail vessel and the mouse was returned to its home cage (all mice handled by tail). A third blood sample (recovery value) was taken 90 min after the start of the stressor, i.e. 75 min after termination of the stressor (mice handled by their assigned handling method).

From the collected blood samples, plasma corticosterone levels were measured as described in detail elsewhere^35^. Briefly, the samples were centrifuged (4,000 g for 10 min at 4 °C), and the plasma was analysed using a corticosterone ELISA kit (EIA 4164, DRG Instruments GmbH, Marburg, Germany). All samples were processed according to the manufacturer’s instructions, with slight modifications detailed elsewhere^34^. All standards, samples and controls were run in duplicates. Intra- and inter-assay coefficients of variance were below 10 and 12%, respectively. The different handling groups were tested in a counterbalanced design. Males and females were tested on consecutive days.

#### Organ weight

After the behavioural and stress physiological tests, the mice were killed by cervical dislocation under deep isoflurane anaesthesia (mixture of 5% isoflurane and ambient air; Forene®, Abb.Vie GmbH & Co. KG, Germany) and adrenal glands and thymus were dissected. The organs were carefully cleared of surrounding fat and subsequently weighed on an analytical scale to the nearest 0.01 mg (Sartorius Competence CPA225D, Göttingen, Germany). The different handling groups were tested in a counterbalanced design. Males and females were tested on consecutive days.

#### Determination of oestrous cycle stage

As the oestrous cycle stage might introduce variability in the outcome of the behavioural and physiological measures^36,37^ (however, for negative results, see Levy and colleagues^38^), vaginal smears were taken immediately after the OF and S-SN to determine the oestrous cycle stage in the female test animals. For this, the mice were placed on the metal grid of the lid of their home cage (handled by their assigned handling method) and lifted gently by the base of the tail to gain access to the vaginal canal. With a sterile inoculation loop (size 1 µl), cells were collected from the vaginal walls and transferred to a microscope slide. After air-drying, slides were Giemsa-stained (Sigma-Aldrich, Darmstadt, Germany), and oestrous cycle stage was quantified as detailed elsewhere^39^.

### Data analysis

To meet the assumptions of parametric analysis, residuals were graphically examined for homoscedasticity and outliers, and the Lillifors corrected Kolmogorov–Smirnov test was applied. We decided to test the two strains separately, as it is known that mouse strains can differ considerably in their behavioural and physiological phenotypes^20–24^. A two-way ANOVA with handling methods and sex as fixed factors was applied to investigate effects on outcomes for the behavioural (Open Field Test, Light–Dark Box test, Sociability and Social Novelty test) and physiological data (fGCM and organ weights). For the AHN a linear mixed model was used with handling method, sex, trial (before/after handling) and stimulus (experimenters’ hand or unfamiliar scent) as fixed factors and cage (mean of the two animals per cage) and session as random factors. A repeated measures ANOVA was calculated for repeated measures (plasma corticosterone measurements and body weight). In case of significant interaction effects, post hoc t-tests were calculated and adjusted using the Bonferroni-Holm method^40^. Data was analysed using IBM SPSS Statistics version 27. For all tests, differences were considered significant if *p* < 0.05.

Graphs were created using GraphPad Prism (version 7.04, GraphPad Software, LLC), and if not noted otherwise, data are depicted as means + standard deviation and individual values (males: circles, females: triangles).

Assessment of the oestrous cycle stage of female mice revealed that receptive and non-receptive stages were not differently represented between groups or measurement time points (Supplementary Table S10). Therefore, it was concluded that potential oestrous cycle effects would balance out and thus were not considered further in the statistical analysis.

## Electronic supplementary material

Below is the link to the electronic supplementary material.

Supplementary Material 1

## Data Availability

The datasets generated and analysed in the current study are available on the repository of Osnabrück University under the following link: 10.26249/FK2/XMQRHC.
