# Supplementary material for: Lack of evidence for a consistent differential impact of tail and tunnel handling on markers of welfare in laboratory mice
Source: Sci Rep. 2025 Jul 1;15:21534. doi: 10.1038/s41598-025-07384-w (PMC12217346; doi:10.1038/s41598-025-07384-w)
Supplement: Supplementary file 1 — Supplementary Material 1 [file 41598_2025_7384_MOESM1_ESM.docx]

**Explanation of sample size deviations**:

According to our *a priori* calculations, we aimed at a sample size of n = 14 mice per group and sex. Animals were excluded from the behavioural tests if they were not tracked correctly by the tracking software and the problem could not be rescued by video re-analysis. Exclusion from the plasma corticosterone and faecal glucocorticoid measurements was due to either obtaining too little blood volume or too few faecal boli. In addition, if technical problems during processing in the hormone assays occurred, the sample was excluded. Organ weights were excluded if the organs were damaged during the dissection or during cleaning from fat tissue prior to weighing. One male B6 mouse was excluded from all analyses as it was found dead (due to unknown reasons) shortly before organ harvesting, as it could not be ruled out that the previously collected behavioural and physiological measurements of this animal might have been affected.

**Supplementary Table S1.** Results of the Open Field test of tail- and tunnel-handled C57BL/6J (B6) and CD-1 mice. Depicted are means and standard deviations (SD). Statistical analysis: Two-way ANOVA with handling method and sex as fixed factors. Significant differences are marked in bold. n = 26 – 27 per group.

| **Open Field test** | | | | | | | |
| --- | --- | --- | --- | --- | --- | --- | --- |
| **B6** | | | | | **ANOVA** | | |
|  | Tail-handled | | Tunnel-handled | | Fixed Factors | | |
| **Behaviour** | **Mean** | **SD** | **Mean** | **SD** | **Handling method** | **Sex** | **Handling method x sex** |
| Total distance (m) | 22.32 | 7.19 | 30.10 | 5.91 | F_1,49_ = 17.735  **p < 0.001** | F_1,49_ = 0.020  p = 0.887 | F_1,49_ = 0.009  p = 0.926 |
| Entries to centre (#) | 13.23 | 5.87 | 14.85 | 4.55 | F_1,49_ = 1.402  p = 0.242 | F_1,49_ = 1.402  p = 0.242 | F_1,49_ = 0.001  p = 0.972 |
| Time in centre (s) | 26.11 | 15.69 | 18.09 | 8.19 | F_1,49_ = 5.114  **p = 0.028** | F_1,49_ = 1.841  p = 0.181 | F_1,49_ = 0.001  p = 0.977 |
| **CD-1** | | | | | **ANOVA** | | |
|  | Tail-handled | | Tunnel-handled | | Fixed Factors | | |
|  | **Mean** | **SD** | **Mean** | **SD** | **Handling method** | **Sex** | **Handling method x sex** |
| Total distance (m) | 34.79 | 8.41 | 35.84 | 7.22 | F_1,48_ = 0.358  p = 0.553 | F_1,48_ = 2.116  p = 0.152 | F_1,48_ = 0.056  p = 0.814 |
| Entries to centre (#) | 26.15 | 7.36 | 29.58 | 6.85 | F_1,48_ = 3.056  p = 0.087 | F_1,48_ = 0.213  p = 0.646 | F_1,48_ = 0.710  p = 0.404 |
| Time in centre (s) | 43.50 | 15.72 | 47.98 | 14.76 | F_1,48_ = 1.117  p = 0.296 | F_1,48_ = 0.040  p = 0.842 | F_1,48_ = 0.565  p = 0.456 |

**Supplementary Table S2.** Results of the Light-Dark Box test of tail- and tunnel-handled C57BL/6J (B6) and CD-1 mice. Depicted are means and standard deviations (SD). Statistical analysis: Statistical analysis: Two-way ANOVA with handling method and sex as fixed factors. Significant differences are marked in bold. n = 26 – 27 per group.

| **Light-Dark Box test** | | | | | | | |
| --- | --- | --- | --- | --- | --- | --- | --- |
| **B6** | | | | | **ANOVA** | | |
|  | Tail-handled | | Tunnel-handled | | Fixed Factors | | |
| **Behaviour** | **Mean** | **SD** | **Mean** | **SD** | **Handling method** | **Sex** | **Handling method x sex** |
| Distance in light compartment (m) | 4.87 | 1.58 | 5.01 | 1.87 | F_1,49_ = 0.185  p = 0.558 | F_1,49_ = 6.430  **p = 0.014** | F_1,49_ = 2.572  p = 0.115 |
| Entries to light compartment (#) | 8.46 | 3.22 | 9.33 | 3.45 | F_1,49_ = 1.173  p = 0.284 | F_1,49_ = 4.517  **p = 0.039** | F_1,49_ = 0.666  p = 0.418 |
| Time in light compartment (s) | 92.15 | 35.58 | 81.80 | 31.06 | F_1,49_ = 1.247  p = 0.270 | F_1,49_ = 1.697  p = 0.199 | F_1,49_ = 2.888  p = 0.096 |
| **CD-1** | | | | | **ANOVA** | | |
|  | Tail-handled | | Tunnel-handled | | Fixed Factors | | |
| **Behaviour** | **Mean** | **SD** | **Mean** | **SD** | **Handling method** | **Sex** | **Handling method x sex** |
| Distance in light compartment (m) | 5.46 | 1.25 | 5.43 | 2.63 | F_1,48_ < 0.001  p = 0.983 | F_1,48_ = 0.832  p = 0.366 | F_1,48_ = 0.123  p = 0.727 |
| Entries to light compartment (#) | 16.58 | 3.68 | 17.00 | 4.60 | F_1,48_ = 0.106  p = 0.746 | F_1,48_ = 0.219  p = 0.642 | F_1,48_ = 0.730  p = 0.397 |
| Time in light compartment (s) | 129.15 | 20.59 | 113.85 | 29.88 | F_1,48_ = 4.629  **p = 0.036** | F_1,48_ = 0.229  p = 0.634 | F_1,48_ = 0.412  p = 0.524 |

**Supplementary Table S3.** Results of the *Sociability Phase* of the Sociability and Social Novelty test of tail- and tunnel-handled C57BL/6J (B6) and CD-1 male mice. Statistical analysis: Two-way ANOVA with handling method and sex as fixed factors. Significant differences are marked in bold. n = 25 – 26 per group.

| **Sociability and Social Novelty test** | | | | | | | |
| --- | --- | --- | --- | --- | --- | --- | --- |
| *Sociability Phase* | | | | | | | |
| **B6** | | | | | **ANOVA** | | |
|  | Tail-handled | | Tunnel-handled | | Fixed Factors | | |
| **Behaviour** | **Mean** | **SD** | **Mean** | **SD** | **Handling method** | **Sex** | **Handling method x sex** |
| Distance (m) | 24.69 | 4,32 | 26.31 | 4.32 | F_1,47_ = 2.757  p = 0.103 | F_1,47_ = 21.628  **p < 0.001** | F_1,47_ = 0.016  p = 0.900 |
| Entries to social partner zone (#) | 79.19 | 23.56 | 84.48 | 16.00 | F_1,47_ = 1.110  p = 0.297 | F_1,47_ = 3.418  p = 0.071 | F_1,47_ = 1.150  p = 0.289 |
| Time in social partner zone (s) | 156.85 | 41.64 | 172.07 | 34.99 | F_1,47_ = 1.918  p = 0.173 | F_1,47_ = 0.109  p = 0.743 | F_1,47_ = 0.031  p = 0.860 |
| Entries to object zone (#) | 76.58 | 20.03 | 73.44 | 19.63 | F_1,47_ = 0.365  p = 0.549 | F_1,47_ = 1.205  p = 0.278 | F_1,47_ = 1.033  p = 0.315 |
| Time in object zone (s) | 124.58 | 31.60 | 121.71 | 32.25 | F_1,47_ = 0.087  p = 0.769 | F_1,47_ = 0.247  p = 0.621 | F_1,47_ = 0.028  p = 0.868 |
| Ratio social partner – object (time) | 0.55 | 0.12 | 0.59 | 0.10 | F_1,47_ = 0.967  p = 0.330 | F_1,47_ = 0.125  p = 0.726 | F_1,47_ = 0.002  p = 0.967 |
| Ratio social partner – object (entries) | 0.50 | 0.12 | 0.54 | 0.09 | F_1,47_ = 1.402  p = 0.242 | F_1,47_ = 0.191  p = 0.664 | F_1,47_ = 1.318  p = 0.257 |
| **CD-1** | | | | | **ANOVA** | | |
|  | Tail-handled | | Tunnel-handled | | Fixed Factors | | |
| **Behaviour** | **Mean** | **SD** | **Mean** | **SD** | **Handling method** | **Sex** | **Handling method x sex** |
| Distance (m) | 28.26 | 4.55 | 32.42 | 5.63 | F_1,48_ = 8.512  **p = 0.005** | F_1,48_ = 0.241  p = 0.626 | F_1,48_ = 0.219  p = 0.642 |
| Entries to social partner zone (#) | 57.85 | 14.57 | 57.88 | 18.42 | F_1,48_ = 0.040  p = 0.841 | F_1,48_ = 7.444  **p = 0.009** | F_1,48_ = 0.168  p = 0.683 |
| Time in social partner zone (s) | 182.65 | 43.86 | 176.72 | 41.17 | F_1,48_ = 0.380  p = 0.540 | F_1,48_ = 1.742  p = 0.193 | F_1,48_ = 2.970  p = 0.091 |
| Entries to object zone (#) | 55.58 | 17.25 | 57.69 | 12.40 | F_1,48_ = 0.258  p = 0.614 | F_1,48_ = 0.007  p = 0.935 | F_1,48_ = 3.672  p = 0.061 |
| Time in object zone (s) | 172.28 | 45.87 | 154.43 | 31.07 | F_1,48_ = 2.303  p = 0.136 | F_1,48_ = 3.684  p = 0.061 | F_1,48_ = 0.004  p = 0.948 |
| Ratio social partner – object (time) | 0.52 | 0.11 | 0.53 | 0.10 | F_1,48_ = 0.144  p = 0.706 | F_1,48_ = 4.168  **p = 0.047** | F_1,48_ = 1.652  p = 0.205 |
| Ratio social partner – object (entries) | 0.56 | 0.11 | 0.51 | 0.11 | F_1,48_ = 1.661  p = 0.204 | F_1,48_ = 2.534  p = 0.118 | F_1,48_ = 0.306  p = 0.583 |

**Supplementary Table S4.** Results of the *Social Novelty Phase* of the Sociability and Social Novelty test of tail- and tunnel-handled C57BL/6J (B6) and CD-1 male mice. Statistical analysis: Two-way ANOVA with handling method and sex as fixed factors. Significant differences are indicated in bold. n = 24 – 26 per group.

| **Sociability and Social Novelty test** | | | | | | | |
| --- | --- | --- | --- | --- | --- | --- | --- |
| *Social Novelty Phase* | | | | | | | |
| **B6** | | | | | **ANOVA** | | |
|  | Tail-handled | | Tunnel-handled | | Fixed Factors | | |
| **Behaviour** | **Mean** | **SD** | **Mean** | **SD** | **Handling method** | **Sex** | **Handling method x sex** |
| Distance (m) | 19.63 | 4.67 | 18.56 | 3.55 | F_1,47_ = 0.850  p = 0.361 | F_1,47_ = 12.136  **p = 0.001** | F_1,47_ = 0.136  p = 0.714 |
| Entries to familiar social partner zone (#) | 50.77 | 11.80 | 54.84 | 15.88 | F_1,47_ = 1.321  p = 0.256 | F_1,47_ = 4.347  **p = 0.043** | F_1,47_ = 0.478  p = 0.493 |
| Time in familiar social partner zone (s) | 100.42 | 30.08 | 131.17 | 48.51 | F_1,47_ = 7.063  **p = 0.011** | F_1,47_ = 0.066  p = 0.799 | F_1,47_ = 0.170  p = 0.682 |
| Entries to novel social partner zone (#) | 75.58 | 16.84 | 69.40 | 15.79 | F_1,47_ = 1.871  p = 0.178 | F_1,47_ = 2.682  p = 0.108 | F_1,47_ = 0.360  p = 0.540 |
| Time in novel social partner zone (s) | 176.83 | 30.59 | 159.21 | 44.14 | F_1,47_ = 2.629  p = 0.112 | F_1,47_ = 0.115  p = 0.736 | F_1,47_ = 0.049  p = 0.825 |
| Ratio familiar social partner – novel social partner (time) | 0.64 | 0.08 | 0.55 | 0.15 | F_1,47_ = 7.073  **p = 0.011** | F_1,47_ = 0.185  p = 0.669 | F_1,47_ = 0.141  p = 0.709 |
| Ratio familiar social partner – novel social partner (entries) | 0.60 | 0.08 | 0.56 | 0.12 | F_1,47_ = 1.748  p = 0.193 | F_1,47_ = 0.121  p = 0.730 | F_1,47_ = 0.748  p = 0.392 |
| **CD-1** | | | | | **ANOVA** | | |
|  | Tail-handled | | Tunnel-handled | | Fixed Factors | | |
| **Behaviour** | **Mean** | **SD** | **Mean** | **SD** | **Handling method** | **Sex** | **Handling method x sex** |
| Distance (m) | 21.01 | 3.26 | 23.93 | 6.31 | F_1,45_ = 3.632  p = 0.063 | F_1,45_ = 0.035  p = 0.852 | F_1,45_ = 0.795  p = 0.377 |
| Entries to familiar social partner zone (#) | 37.00 | 11.19 | 40.08 | 15.71 | F_1,45_ = 0.444  p = 0.509 | F_1,45_ = 0.919  p = 0.343 | F_1,45_ = 0.014  p = 0.908 |
| Time in familiar social partner zone (s) | 131.27 | 47.58 | 130.96 | 40.84 | F_1,45_ < 0.001  p = 0.999 | F_1,45_ = 0.148  p = 0.703 | F_1,45_ = 1.049  p = 0.311 |
| Entries to novel social partner zone (#) | 52.79 | 12.78 | 48.92 | 12.99 | F_1,45_ = 1.778  p = 0.189 | F_1,45_ = 0.131  p = 0.719 | F_1,45_ = 8.372  **p = 0.006** |
| Time in novel social partner zone (s) | 198.80 | 40.57 | 171.81 | 28.05 | F_1,45_ = 7.771  **p = 0.008** | F_1,45_ = 0.051  p = 0.822 | F_1,45_ = 1.562  p = 0.218 |
| Ratio familiar social partner – novel social partner (time) | 0.61 | 0.11 | 0.57 | 0.10 | F_1,45_ = 1.499  p = 0.227 | F_1,45_ = 0.002  p = 0.967 | F_1,45_ = 2.844  p = 0.099 |
| Ratio familiar social partner – novel social partner (entries) | 0.57 | 0.11 | 0.55 | 0.11 | F_1,45_ = 0.340  p = 0.563 | F_1,45_ = 2.013  p = 0.163 | F_1,45_ = 0.883  p = 0.352 |

**Supplementary Table S5.** Body weight (g) of tail- and tunnel-handled C57BL/6J (B6) and CD-1 mice throughout the experiment**.** Data for experimental weeks 10, 15, 17 and 18 are presented as means and SD. Statistics: Repeated-measures ANOVA (with handling method and sex as fixed factors) over the experimental weeks (age). Significant differences are indicated in bold; n = 26-28 per group.

| **B6** | | | | | | | | |
| --- | --- | --- | --- | --- | --- | --- | --- | --- |
| Body weight (g) |  | | | | | | | |
|  | Tail-handled | | Tunnel-handled | | **Repeated-measures ANOVA** | | | |
| **Experimental week** | **Mean** | **SD** | **Mean** | **SD** | **Age** | **Age x handling** | **Age x sex** | **Age x handling x sex** |
| Week 10 | 23.19 | 3.13 | 22.95 | 3.10 | F_3,147_ = 308.727 **p < 0.001** | F_3,147_ = 1.081 p = 0.359 | F_3,147_ = 18.637 **p < 0.001** | F_3,147_ = 3.510 **p = 0.017** |
| Week 15 | 25.29 | 3.66 | 25.30 | 3.66 |  |  |  |  |
| Week 17 (following OF) | 25.59 | 3.56 | 25.59 | 3.56 |  |  |  |  |
| Week 18 (following S-SN) | 25.63 | 3.50 | 25.63 | 3.50 |  |  |  |  |
| **CD-1** | | | | | | | | |
|  | Tail-handled | | Tunnel-handled | | **Repeated-measures ANOVA** | | | |
| **Experimental week** | **Mean** | **SD** | **Mean** | **SD** | **Age** | **Age x handling** | **Age x sex** | **Age x handling x sex** |
| Week 10 | 36.07 | 5.62 | 36.62 | 4.32 | F_3,144_ = 86.607 **p < 0.001** | F_3,144_ = 0.295 p = 0.829 | F_3,144_ = 3.069 **p = 0.030** | F_3,144_ = 0.546 p = 0.652 |
| Week 15 | 39.27 | 6.66 | 40.02 | 4.65 |  |  |  |  |
| Week 17 (following OF) | 39.67 | 6.77 | 40.54 | 5.30 |  |  |  |  |
| Week 18 (following S-SN) | 39.72 | 6.81 | 40.92 | 5.52 |  |  |  |  |

**Supplementary Table S6.** Faecal glucocorticoid metabolite (fGCM) concentrations (ng/0.05g dry faeces) of tail and tunnel-handled C57BL/6J (B6) and CD-1 mice in response to the assigned handling method (tail or tunnel). Eight hours after handling, faecal samples voided within a one-hour time window were collected. Data are presented as means and SD. Statistical analysis: Two-way ANOVA with handling method and sex as fixed factors was performed to investigate the influence of the handling method in each strain. Significant differences are indicated in bold; n = 23 - 26 per group.

| **fGCM concentration (**ng/0.05g dry faeces) | | | | | | | |
| --- | --- | --- | --- | --- | --- | --- | --- |
| **B6** | | | | | **ANOVA** | | |
|  | Tail-handled | | Tunnel-handled | | Fixed Factors | | |
|  | **Mean** | **SD** | **Mean** | **SD** | **Handling method** | **Sex** | **Handling method x sex** |
| fGCM concentration | 31.09 | 14.18 | 40.48 | 15.70 | F_1,45_ = 4.894  **p = 0.032** | F_1,45_ = 13.108  **p = 0.001** | F_1,4_ = 1.561  p = 0.218 |
| **CD-1** | | | | | **ANOVA** | | |
|  | Tail-handled | | Tunnel-handled | | Fixed Factors | | |
|  | **Mean** | **SD** | **Mean** | **SD** | **Handling method** | **Sex** | **Handling method x sex** |
| fGCM concentration | 34.48 | 26.67 | 51.30 | 51.18 | F_1,45_ = 2.931  p = 0.094 | F_1,45_ = 10.863  **p = 0.002** | F_1,45_ = 1.940  p = 0.171 |

**Supplementary Table S7.** Initial, reaction and recovery plasma corticosterone concentrations (ng/ml) of tail and tunnel-handled C57BL/6J and CD-1 mice assessed in the Stress Reactivity Test (SRT). Data are presented as means and SD. A repeated-measures ANOVA over the three blood sampling time points (Initial, Reaction, Recovery) with handling method and sex as fixed factors was performed to investigate the influence of the handling method in each strain; Significant differences are indicated in bold; n = 20 – 27 per group

| **Plasma corticosterone levels** (ng/ml) | | | | | | | | |
| --- | --- | --- | --- | --- | --- | --- | --- | --- |
| **B6** | | | | | **Repeated-measured ANOVA** | | | |
|  | Tail-handled | | Tunnel-handled | | Fixed Factors | | | |
| **Sampling time point** | **Mean** | **SD** | **Mean** | **SD** | **Sampling time point (STP)** | **STP x handling method** | **STP x sex** | **STP x Handling method x sex** |
| Initial | 44.74 | 42.20 | 41.34 | 29.72 | F_1.8,86.5_ = 204.781  **p < 0.001** | F_1.8,86.5_ = 2.372  p = 0.106 | F_1.8,86.5_ = 0.291  p = 0.721 | F_1.8,86.5_ = 0.140  p = 0.870 |
| Reaction | 253.34 | 65.53 | 236.62 | 49.66 |  |  |  |  |
| Recovery | 105.17 | 61.35 | 131.74 | 92.59 |  |  |  |  |
| **CD-1** | | | | | **Repeated-measured ANOVA ANOVA** | | | |
|  | Tail-handled | | Tunnel-handled | | Fixed Factors | | | |
| **Sampling time point** | **Mean** | **SD** | **Mean** | **SD** | **Sampling time point (STP)** | **STP x handling method** | **STP x sex** | **STP x Handling method x sex** |
| Initial | 56.19 | 69.25 | 39.85 | 36.85 | F_1.3,51.7_ = 80.070  **p < 0.001** | F_1.3,51.7_ = 0.535  p = 0.517 | F_1.3,51.7_ = 5.602  **p = 0.005** | F_1.3,51.7_ = 0.342  p = 0.623 |
| Reaction | 341.53 | 103.80 | 350.21 | 118.09 |  |  |  |  |
| Recovery | 182.94 | 151.89 | 217.50 | 161.59 |  |  |  |  |

**Supplementary Table S8.** Organ weights relative to body weight (BW) for adrenal glands and thymus as a markers for chronic stress in tail- and tunnel-handled C57BL/6J (B6) and CD-1 mice. Data are presented as means and SD. A two-way ANOVA with handing method and sex as fixed factors was performed to investigate the influence of handling method in each strain; n = 21 - 22 per group.

| **Organ weight** (mg/g BW) | | | | | | | |
| --- | --- | --- | --- | --- | --- | --- | --- |
| **B6** | | | | | **ANOVA** | | |
|  | Tail-handled | | Tunnel-handled | | Fixed Factors | | |
| **Organ** | **Mean** | **SD** | **Mean** | **SD** | **Handling method** | **Sex** | **Handling method x sex** |
| Mean of left and right adrenal glands | 0.079 | 0.027 | 0.087 | 0.030 | F_1,37_ = 0.006  p = 0.937 | F_1,37_ = 99.842  **p < 0.001** | F_1,37_ = 0.060  p = 0.807 |
| Thymus | 1.492 | 0.451 | 1.435 | 0.417 | F_1,40_ = 0.728  p = 0.399 | F_1,40_ = 44.516  **p < 0.001** | F_1,40_ = 0.178  p = 0.675 |
| **CD-1** | | | | | **ANOVA** | | |
|  | Tail-handled | | Tunnel-handled | | Fixed Factors | | |
| **Organ** | **Mean** | **SD** | **Mean** | **SD** | **Handling method** | **Sex** | **Handling method x sex** |
| Mean of left and right adrenal glands | 0.096 | 0.032 | 0.092 | 0.031 | F_1,32_ = 0.071  p = 0.792 | F_1,32_ = 66.653  **p < 0.001** | F_1,32_ = 0.048  p = 0.827 |
| Thymus | 0.838 | 0.226 | 0.858 | 0.355 | F_1,39_ = 0.028  p = 0.869 | F_1,37_ = 38.983  **p < 0.001** | F_1,48_ = 2.855  p = 0.099 |

**Supplementary Table S9.** Handling times per cage (two mice per cage) in seconds (means ± SD), starting at postnatal week (PNW) 6 of male and female B6 and CD-1 mice. Please note that there is no handling time data available for PNW 10 and 15, as at these time points the animals were weighed in addition to being handled.

|  | **B6** | | **CD-1** | |
| --- | --- | --- | --- | --- |
| **PNW** | **Tail handling (sec)** | **Tunnel handling (sec)** | **Tail handling (sec)** | **Tunnel handling (sec)** |
| **6** | 10.85 ± 3.00 | 26.64 ± 11.13 | 10.17 ± 1.94 | 86.16 ± 59.92 |
| **7** | 14.69 ± 7.63 | 26.71 ± 9.03 | 9.80 ± 3.43 | 33.73 ± 17.40 |
| **8** | 10.69 ± 2.93 | 36.00 ± 13.45 | 9.67 ± 2.55 | 45.60 ± 61.20 |
| **9** | 11.77 ± 4.11 | 27.79 ± 14.89 | 8.00 ± 1.25 | 20.71 ± 14.54 |
| **11** | 11.69 ± 3.86 | 55.36 ± 19.85 | 8.47 ± 1.88 | 26.87 ± 10.39 |
| **12** | 7.62 ± 1.89 | 40.71 ± 25.64 | 8.00 ± 1.89 | 28.27 ± 19.65 |
| **13** | 9.54 ± 2.67 | 48.31 ± 29.78 | 9.53 ± 3.56 | 28.67 ± 14.97 |
| **14** | 7.54 ± 1.76 | 58.08 ± 34.64 | 8.53 ± 1.92 | 35.87 ± 24.76 |
| **Mean:** | **10.55 ± 4.34** | **39.71 ± 23.81** | **8.93 ± 2.50** | **34.47 ± 32.83** |

**Supplementary Table S10.** Oestrous cycle stages of female C57BL/6J (B6) and CD-1 mice divided into number of receptive (oestrous and pro-oestrous) and non-receptive (dioestrous and metoestrous) individuals at different time points. Statistical analysis: Chi-Square test. No significant differences in the distribution of receptive and non-receptive mice were detected between groups at the different time points. Abbreviations: OF test, Open Field test, LD test, Light-Dark Box test, S-SN test, Sociability and Social Novelty test.

|  | OF test | LD test | S-SN test |
| --- | --- | --- | --- |
| **B6 tail-handled females** | | | |
| receptive | 7 | 8 | 6 |
| non-receptive | 5 | 4 | 6 |
| **B6 tunnel-handled females** | | | |
| receptive | 8 | 7 | 7 |
| non-receptive | 6 | 7 | 5 |
| Statistical analysis | Χ(1) = 0.004, p = 0.951 | Χ(1) = 0.735, p = 0.391 | Χ(1) = 0.168, p = 0.682 |
| **CD-1 tail-handled females** | | | |
| receptive | 7 | 6 | 7 |
| non-receptive | 7 | 8 | 7 |
| **CD-1 tunnel-handled females** | | | |
| receptive | 5 | 8 | 6 |
| non-receptive | 7 | 4 | 6 |
| Statistical analysis | Χ(1) = 0.181, p = 0.671 | Χ(1) = 1.474, p = 0.225 | Χ(1) < 0.001, p > 0.999 |
